# Lesser than diabetes hyperglycemia in pregnancy is related to perinatal mortality: a cohort study in Brazil

**DOI:** 10.1186/1471-2393-11-92

**Published:** 2011-11-11

**Authors:** Eliana M Wendland, Bruce B Duncan, Sotero S Mengue, Maria I Schmidt

**Affiliations:** 1 Post-Graduate Program in Epidemiology, Federal University of Rio Grande do Sul, Porto Alegre, Brazil

## Abstract

**Background:**

Gestational diabetes related morbidity increases along the continuum of the glycemic spectrum. Perinatal mortality, as a complication of gestational diabetes, has been little investigated. In early studies, an association was found, but in more recent ones it has not been confirmed. The Brazilian Study of Gestational Diabetes, a cohort of untreated pregnant women enrolled in the early 1990's, offers a unique opportunity to investigate this question. Thus, our objective is to evaluate whether perinatal mortality increases in a continuum across the maternal glycemic spectrum.

**Methods:**

We prospectively enrolled and followed 4401 pregnant women attending general prenatal care clinics in six Brazilian state capitals, without history of diabetes outside of pregnancy, through to birth, and their offspring through the early neonatal period. Women answered a structured questionnaire and underwent a standardized 2-hour 75-g oral glucose tolerance test (OGTT). Obstetric care was maintained according to local protocols. We obtained antenatal, delivery and neonatal data from hospital records. Odds ratios (OR) were estimated using logistic regression.

**Results:**

We ascertained 97 perinatal deaths (67 fetal and 31 early neonatal). Odds of dying increased according to glucose levels, statistically significantly so only for women delivering at gestational age ≥34 weeks (p < 0.05 for glycemia-gestational age interaction). ORs for a 1 standard deviation difference in glucose, when analyzed continuously, were for fasting 1.47 (95% CI 1.12, 1.92); 1-h 1.55 (95% CI 1.15, 2.07); and 2-h 1.53 (95% CI 1.15, 2.02). The adjusted OR for IADPSG criteria gestational diabetes was 2.21 (95% CI 1.15, 4.27); and for WHO criteria gestational diabetes, 3.10 (95% CI 1.39, 6.88).

**Conclusions:**

In settings of limited detection and treatment of gestational diabetes mellitus, women across a spectrum of lesser than diabetes hyperglycemia, experienced a continuous rise in perinatal death with increasing levels of glycemia after 34 weeks of pregnancy. Current GDM diagnostic criteria identified this increased risk of mortality.

## Background

Gestational diabetes is generally defined as a state of glucose intolerance detected during pregnancy, but the level of hyperglycemia used in its definition varies remarkably around the world, as does its prevalence [1]. Gestational diabetes is an established risk factor for adverse maternal outcomes such preeclampsia and future type 2 diabetes, as well as neonatal outcomes such as macrosomia, hypoglycemia and birth injuries [2-6].

Perinatal mortality was reported to be higher among women with gestational diabetes in some initial studies [7,8]. However, the HAPO Study, a large multi-country cohort study conducted between 2000 and 2006, found no association between glucose levels and perinatal mortality [6]. In fact, this association was also not found in an additional large, retrospective cohort study conducted in a developing country [9].

Worldwide, an estimated 6.3 million perinatal deaths, of which 57% are fetal deaths, occur annually [10]. The contribution of gestational diabetes to perinatal mortality is controversial, particularly with respect to milder degrees of hyperglycemia [6-9]. We have previously reported greater perinatal death for women meeting American Diabetes Association criteria for gestational diabetes in the Brazilian Study of Gestational Diabetes (Estudo Brasileiro de Diabetes Gestacional; EBDG) [3]. This large cohort study was conducted in the early 1990's, a time during which the screening and treatment for gestational diabetes was controversial and not widespread in Brazil.

The aim of this report is to describe, in the EBDG population, the association of perinatal death with maternal glucose levels when examined across the continuum of glucose levels and additionally using current diagnostic criteria.

## Methods

The Brazilian Study of Gestational Diabetes investigated a cohort of Brazilian pregnant women enrolled between 1991 and 1995, allowing analysis of untreated women with hyperglycemia below that diagnostic of diabetes outside of pregnancy [3]. We consecutively recruited 5564 women aged 20 or more years, with no history of diabetes outside pregnancy, who attended general prenatal care clinics in six Brazilian state capitals (Porto Alegre, São Paulo, Rio de Janeiro, Salvador, Fortaleza and Manaus). Of these, 4998 underwent a 2-hour 75-g OGTT between their 24th and 28th weeks of pregnancy following standard procedures previously described [3]. After excluding 21 women meeting diagnostic criteria for diabetes outside of pregnancy, 2 with gestational diabetes treated with insulin, 49 twin pregnancies, one with questionable birth weight and 524 with birth less than 28 weeks of pregnancy, we examined 4401 pregnancies. The study protocol was approved by each local institutional ethics committee, and women consented to participate after being informed about the nature of the study.

All women answered a structured questionnaire at enrollment and were followed through delivery and in the postpartum period through chart review. We calculated pre-pregnancy BMI using self-reported weight and measured height. All patients received routine obstetric care by their physicians. We obtained neonatal data from hospital records. We defined perinatal death as a death of offspring occurring after 28 weeks of pregnancy or up to seven complete days of life [10], and macrosomia as a birthweight at or above the gestational age-specific (by week) 90th percentile of the study sample, as previously described [3].

For descriptive analyses, we used glucose categories previously defined to permit comparison with previous studies [6]. The Cochrane-Armitage test was used to evaluate statistical significance of trends [11]. We employed analysis of covariance to compare adjusted means of glycemia between groups. We used logistic regression to estimate odds ratios for perinatal death associated with a 1 standard deviation difference in fasting, 1-hour and 2-hour plasma glucose levels adjusted for covariates. Selection of variables for inclusion in regression models was based on their relationship with outcomes. Due to the small number of events, final models included only variables which led to the largest change in the odds ratio Similar regression analyses were done having the categorical variable gestational diabetes as the main exposure. Two definitions were used, that of the World Health Organization (WHO) [12] and that of the International Association of Diabetes and Pregnancy Study Groups (IADSPG) [13]. A significant difference was defined as a p value < 0.05.

## Results

Clinical characteristics of the study sample, including plasma glucose means for fasting, 1-hour and 2-hour moments of the 75 g OGTT are given in Table 1.

**Table 1:** Demographic and clinical characteristics of study subjects.

	*Mean*	*SD*	*Minimum*	*Maximum*
Age (years)	27.8	5.5	20.0	48.0
Pre-pregnancy body mass index (kg/m^2^)	23.4	4.1	12.9	52.9
Education (years)	7.8	3.7	0	16.0
Birth weight (g)	3207.0	558.2	650.0	5650.0
Gestational age at delivery (weeks)	38.9	2.2	28.0	44.8
Plasma glucose (mg/dl)				
Fasting	81.7	10.6	43.0	124.0
1-hour	121.7	27.9	41.0	245.0
2-hour	103.7	22.7	33.0	199.0
Gestational age at delivery (weeks)	38.9	2.2	28.0	44.8
Birth weight (g)	3207.0	558.2	650	5650

SD = standard deviation

We ascertained 97 perinatal deaths, 66 being fetal and 31 occurring in the early neonatal period. The perinatal mortality rate estimated from week 28 of pregnancy to 1 week postpartum was 22.0/1000. A total of 38 (39%) deaths occurred before week 34 of pregnancy and 59 (61%), thereafter. Macrosomia was observed in 3 (7.5%) perinatal deaths and in 376 (9.7%) of those surviving the perinatal period (p = 0.21); small for gestational age in 16 (39.0%) deaths and 368 (9.9) survivors (p < 0.001).

On average, mothers of offspring with perinatal death had one year less education (6.9 vs. 7.8; p = 0.02) than those with surviving offspring, but similar mean age (28.3 vs. 27.8 years; p = 0.38), pre-pregnancy BMI (23.2 vs. 23.4; p = 0.61), skin color (white 36.1% vs. 44.7%; p = 0.09), parity (nulliparity 24.1% vs. 30.8%; p = 0.2) and rates of cesarean section (40.4% vs. 37.8%; p = 0.6).

Over the gestational age span evaluated, fasting (83.2 mg/d vs. 81.5 mg/dl; p = 0.17) and 1-hour (124.8 mg/dl vs. 118.9mg/dl; p = 0.07) glucose values, adjusted for maternal age, skin color, years of education, parity and pre-pregnancy body mass index, did not differ for those with and without perinatal death. However, the adjusted mean 2-hour glucose level was higher in women suffering perinatal death (110.2 mg/dl vs. 104.2 mg/dl; p = 0.03).

Similarly, greater odds of perinatal death, adjusted through logistic regression for race, parity, maternal age, pre-pregnancy BMI and years of education, were found only for higher glucose levels at 2-h post load: OR 1.28; 95% CI 1.02, 1.59) for a 1 standard deviation difference in glucose level (Table 2). The association between glucose and perinatal death varied depending upon the gestational period in which it was evaluated (p < 0.05 for the glycemia-gestational age interaction for all 3 glucose tolerance moments). For events occurring at 34 weeks or later, a one standard deviation increase in glucose was associated with greater odds of dying (Table 2): fasting 1.47 (95% CI 1.12, 1.92); 1-h 1.55 (95% CI 1.15, 2.07) and 2-h 1.53 (95% CI 1.15, 2.02).

**Table 2:** Association of perinatal mortality* with levels of fasting, and 1-hour and 2-hour glucose post 75 g load for women delivering at different gestational periods

	*Fasting*		*1-hour*		*2-hour*	
	OR	95% CI	OR	95% CI	OR	95% CI
Total sample						
Model 1	1.11	0.91,1.35	1.13	0.93,1.38	1.14	0.93,1.38
Model 2	1.16	0.93,1.45	1.25	0.99,1.57	1.28	1.02,1.59
Delivery between 28 and 33 weeks						
Model 1	0.93	0.64,1.34	0.87	0.59,1.27	0.82	0.56,1.21
Model 2	0.83	0.54,1.29	0.85	0.54,1.34	0.97	0.62,1.52
Delivery ≥ 34 weeks						
Model 1	1.26	0.99,1.59	1.30	1.02,1.67	1.37	1.07,1.74
Model 2	1.47	1.12,1.92	1.55	1.15,2.07	1.53	1.15,2.02

*adjusted through logistic regression for:

Model 1: maternal age and race/ethnicity

Model 2: maternal age, race/ethnicity, parity, pre-pregnancy BMI, years of education

**Figure 1:**
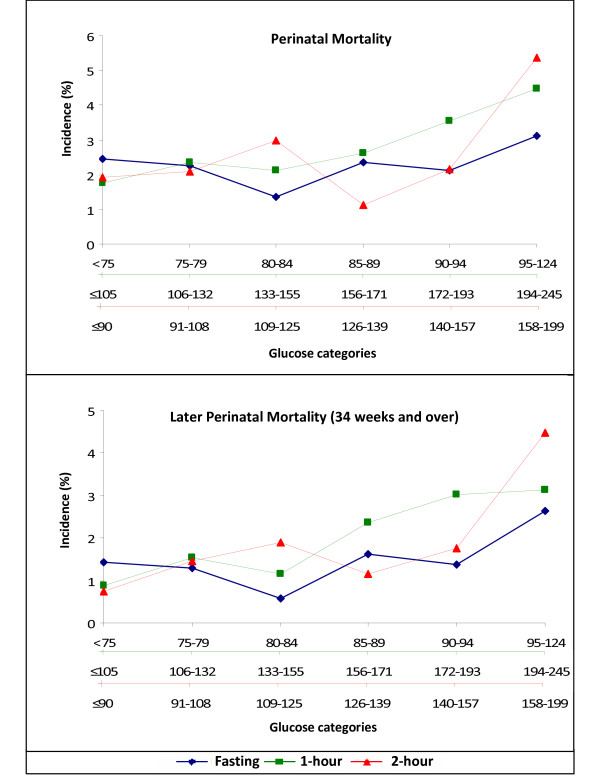
**Incidence of perinatal death according to glucose categories for fasting, 1-hour and 2-hour moments of the 75 gr oral glucose tolerance test**. Exact Cochrane-Armitage test for trend for overall perinatal mortality (fasting = 0.30; 1-hour = 0.05; 2-hour = 0.13) and for later perinatal mortality (fasting = 0.06; 1-hour = 0.01; 2-hour <0.01). To convert mg/dl to mmol/L divide by 18.02.

To evaluate to what extent this association occurs across the continuum of glucose levels, we plotted the crude incidence of perinatal death according to glucose categories (Figure 1). As seen in the upper panel (gestational age at delivery ≥ 28 weeks), incidence of perinatal death increased according to glucose categories, but linear trend was not statistically significant. For women with delivery at gestational age ≥ 34 weeks (lower panel), the incidence of perinatal death increased with increasing glucose values at all moments of the OGTT: from 1.4% to 2.6% comparing the lowest (<75 mg/dl) to the highest (≥95 mg/dl) fasting glucose; from 0.9% to 3.1% comparing the lowest (≤105 mg/dl) to highest 1-hour glucose (≥194 mg/dl) and from 0.7% to 4.5% comparing the lowest (≤90 mg/dl) to highest 2-hour glucose (≥158 mg/dl). Linear trends were statistically significant for the 1-hour glucose (p = 0.01) and the 2-hour (p < 0.001) glucose categories. Additionally, when considering only this latter period, mean plasma glucose values (mg/dl), adjusted for maternal age, skin color, years of education, parity and pre-pregnancy body mass index, were higher in women with a perinatal death for all glucose measurements: fasting (85.8 mg/dl vs. 81.5 mg/dl p < 0.05), 1-hour (129.9 mg/dl vs. 118.8 mg/dl; p < 0.01) and 2-hour (114.2 mg/dl vs. 104.4 mg/dl; p < 0.01).

Logistic regression analysis, evaluating the association of perinatal mortality with the IADPSG and the WHO criteria for gestational diabetes, also demonstrated associations only for women delivering after 34 weeks (Table 3). Associations were always somewhat larger with the WHO criteria (adjusted OR 3.10 (95% CI 1.39, 6.88), than with the IADPSG criteria (adjusted OR 2.21 (95% CI 1.15, 4.27).

**Table 3:** Association of perinatal mortality* with different diagnostic criteria for gestational diabetes for women delivering at different gestational periods

	IADSPG		WHO	
	
	OR	95% CI	OR	95% CI
Total sample (n = 4398)				
Model 1	1.38	0.86,2.22	1.46	0.74,2.85
Model 2	1.47	0.85,2.54	2.11	1.06,4.23
Delivery between 28 and 33 weeks (n = 168)				
Model 1	0.75	0.28,1.99	0.82	0.16,4.19
Model 2	0.63	0.19,2.06	1.12	0.20,6.30
Delivery ≥ 34 weeks (n = 4230)				
Model 1	1.78	1.01,3.17	1.98	0.92,4.25
Model 2	2.21	1.15,4.27	3.10	1.39,6.88

*adjusted through logistic regression for: maternal age and race/ethnicity (Model 1) and maternal age, race/ethnicity, parity, pre-pregnancy BMI, years of education (Model 2). IADSPG = International Association of Diabetes and Pregnancy Study Groups (a fasting glucose ≥ 5.1 mmol/L (92 mg/dl); a one hour result of ≥ 10.0 mmol/L (180 mg/dl); or a two hour result of ≥ 8.5 mmol/L (153 mg/dl).

WHO = World Health Organization (a two hour post load glycemia > 7.8 mmol/L (140 mg/dl).

## Discussion

Perinatal mortality is the most serious adverse outcome of pregnancy, but evidence for its association with lesser than diabetes hyperglycemia is less clear. We report excess perinatal mortality risk with increasing glucose values across the spectrum of lesser than diabetes hyperglycemia in late pregnancy in a cohort study conducted at a setting of limited testing for and treatment of gestational diabetes. This greater risk of death was present in women with GDM diagnosed by both the WHO and the IADPGS criteria.

The literature is controversial on this issue. Like ours, some previous studies have shown that glycemic levels are associated with perinatal mortality. O'Sullivan et al. [8] found a 4-fold increase in perinatal mortality rate in women with untreated gestational diabetes. Pettit et al. [7] reported a direct association between glucose levels from a 75-g 2-hour glucose challenge and perinatal mortality in Pima Indians. However, both of these studies included women with more severe hyperglycemia.

A more recent major study, HAPO, did not find an association in unadjusted logistic analysis [6]. Several differences between the HAPO study design and ours could explain this difference. First, obstetric care has improved dramatically around the world. This has led to major decreases in perinatal mortality from the early 1990s, when EBDG was performed, to the early years of the current decade, when HAPO was undertaken. The perinatal mortality rate in our study, 22/1000, was similar to the rates existing in Brazil at that time [14], and much higher than that reported in the HAPO study (5.6/1000). Improved obstetric care, for example, fetal monitoring, and treatment of metabolic instability in early life and complications of macrosomia, can counterbalance some of the risk resulting from gestational diabetes. Second, women with a fasting glucose higher then 105 mg/dl at testing or at any point during follow-up were excluded from HAPO analyses, producing a sample with milder hyperglycemia than that of EBDG.

Ramtoola et al. [9] found slightly lower perinatal mortality (22/1000) with gestational diabetes as defined by the World Health Organization diagnostic criteria (impaired glucose tolerance) than that of the background population of Mauritian women (26/1000). However, the prevalence of gestational diabetes, ascertained without universal screening, was only 1% in their study (184 women, 98 characterized as having impaired glucose tolerance and 86 as having diabetes, out of approximately 4500 annual births in each of the 4 years studied). This prevalence contrasts with that of 7.8% with universal screening in our study [3]. The lower mortality they found could thus be due to unexplained confounding resulting from non-universal screening. In previous analyses of our Brazilian cohort [3], we found a small, though not statistically significant, increase in perinatal mortality [RR = 1.66; 95% CI 0.91, 2,96] when using the WHO criteria. As our previous study reporting mortality associations [3] analyzed additional outcomes, its exclusions were somewhat different, resulting in associations of a slightly different magnitude than those reported here.

The main strength of our study is its prospective cohort design. Moreover, given the controversial nature of the association between lesser than diabetes hyperglycemia and perinatal death, our study was able to demonstrates that, in settings of low detection and treatment of gestational diabetes, positive associations of glucose values with perinatal death are more evident in late pregnancy, a period when deaths associated to prematurity complications are improbable [15]. Additionally, we demonstrate that both current GDM diagnostic criteria detect women having this increased risk.

Our study has some limitations. Instability is present because of the small number of perinatal deaths. This small number also hindered our ability to evaluate the presence of a glucose threshold for increased perinatal mortality. However, at glucose categories 5 (for fasting 90-94 mg/dl, for 1 h 172-193 mg/dl, and 2 h 140-157 mg/dl) the increase in risk is evident, regardless of the period of pregnancy (Figure 1). Additionally, as we did not retest women for GDM after week 28, it is also possible that the associations here described underestimate the true associations, as deaths in women who only developed GDM after week 28 will be considered as not associated with GDM, thus diluting the association. Finally, other, unmeasured risk factors for perinatal death could confound the reported associations. In this regard, the additional inclusion of preeclampsia in the logistic model did not materially alter the association found (data not shown).

## Conclusions

Our study shows an association between glycemia and perinatal mortality in late pregnancy in women not treated for gestational diabetes and receiving standard obstetric care in the early 1990s in Brazil. Both the WHO and the IADPSG criteria detected women having this increased risk. Much of the world's population lives in settings with a level of perinatal mortality and quality of obstetric care similar to or worse than that present in our study. Additionally, women in these settings, predominantly in low and middle-income countries, are suffering the nutritional transition, which increases the risk for hyperglycemia in pregnancy. Thus, gestational diabetes may continue to be an important cause of perinatal mortality for many of the world's childbearing women.

## Competing interests

The authors declare that they have no competing interests.

## Authors' contributions

EMW performed the statistical analysis and drafted the manuscript; MIS participated in design and coordination of the study and manuscript preparation; BBD participated in the design of the study and manuscript preparation. SSM participated in the in the design of the study and data management. All authors read and approved the final manuscript.

## Pre-publication history

The pre-publication history for this paper can be accessed here:

http://www.biomedcentral.com/1471-2393/11/92/prepub
